# Dietary iron intake in the first 4 months of infancy and the development of type 1 diabetes: a pilot study

**DOI:** 10.1186/1758-5996-2-58

**Published:** 2010-09-20

**Authors:** Ambika P Ashraf, Nancy B Eason, Edmond K Kabagambe, Josna Haritha, Sreelatha Meleth, Kenneth L McCormick

**Affiliations:** 1 Department of Pediatrics/Division of Pediatric Endocrinology and Metabolism, The Children's Hospital, University of Alabama at Birmingham, USA; 2 Alabama A & M University, USA (at the time of study; 3 The Department of Epidemiology, University of Alabama at Birmingham, USA; 4 UAB school of Medicine, University of Alabama at Birmingham, USA; 5 The Division of Preventive Medicine, University of Alabama at Birmingham, Alabama, USA

## Abstract

**Aims:**

To investigate the impact of iron intake on the development of type 1 diabetes (T1DM).

**Methods:**

Case-control study with self-administered questionnaire among families of children with T1DM who were less than 10 years old at the time of the survey and developed diabetes between age 1 and 6 years. Data on the types of infant feeding in the first 4 months of life was collected from parents of children with T1DM (n = 128) and controls (n = 67) <10 years old. Because some cases had sibling controls, we used conditional logistic regression models to analyze the data in two ways. First we performed a case-control analysis of all 128 cases and 67 controls. Next, we performed a case-control analysis restricted to cases (n = 59) that had a sibling without diabetes (n = 59). Total iron intake was modeled as one standard deviation (SD) increase in iron intake. The SD for iron intake was 540 mg in the total sample and 539 mg in the restricted sample as defined above.

**Results:**

The median (min, max) total iron intake in the first 4 months of life was 1159 (50, 2399) mg in T1DM cases and 466 (50, 1224) mg among controls (*P *< 0.001). For each one standard deviation increase in iron intake, the odds ratio (95% confidence interval) for type 1 diabetes was 2.01 (1.183, 3.41) among all participants (128 cases and 67 controls) while it was 2.26 (1.27, 4.03) in a restricted sample of T1 D cases with a control sibling (59 cases and 59 controls) in models adjusted for birth weight, age at the time of the survey, and birth order.

**Conclusion:**

In this pilot study, high iron intake in the first 4 months of infancy is associated with T1DM. Whether iron intake is causal or a marker of another risk factor warrants further investigation.

## Background

The incidence of type 1 diabetes (T1DM) is increasing worldwide and the age of onset is shifting downwards with a significant number of cases occurring between birth and 4 years of age [1-4]. Although the causes for increased incidence and early onset of T1DM are uncertain [1], a number of risk factors associated with induction of autoimmunity in the genetically susceptible individuals have been suggested. These include viral infections (especially enterovirus), stressful life events, vaccinations, early exposure to milk protein, such as bovine insulin, casein and other proteins, early introduction of cereal to infants, low serum vitamin D levels and premature exposure of the gut immune system to foreign proteins [5-19]. The role of environmental determinants in initiating the autoimmunity cascade has also been suggested by studies in discordant/concordant twins [20,21]. Prolonged breast feeding may protect against type 1 diabetes [22,23]. Timing of introduction of solids/cereals was found to influence the risk of developing islet autoimmunity whereas breastfeeding duration/introduction of cow's milk were not statistically associated with the risk factor for developing islet auto antibodies [11,24]. There are no major associations reported between breast feeding and risk of T1DM [11,25,26].

Iron, a major supplement in fortified baby formula, could play a role in modifying the risk for T1DM given that studies in adults have suggested an association between iron burden and T2DM. During the first 4 months of life, there is an increased breakdown of fetal erythrocytes leading to increased amounts of circulating iron that are subsequently stored in the parenchymal cells of liver and reticuloendothelial macrophages [27,28]. The estimated iron requirement for the term infant for the expansion of the erythrocyte mass and to maintain adequate stores is 1 mg/kg/day [27,29]. Iron absorption for term infants fed cow's milk formulas varies from 4-12% depending on a variety of factors including age of the infant, reticulocyte count, body iron status, and composition of the baby formula (e.g., calcium concentration and added ascorbic acid). Absorption levels are even lower with soy milk formula. Studies using isotopically labeled iron reveal that the bioavailability of iron in infant formulas is approximately 11% [30] and 11.3% in premature and term babies, respectively [31]. Compared to breast milk, from which approximately 50% of the iron is absorbed [27,31,32], only about 19% of the iron in baby formula is absorbed [33]. In vivo, recombinant human lactoferrin added to the infant formula attenuated iron mediated free radical formation and lipid peroxidation [34]. Thus, lack of breast milk lactoferrin may predispose the formula fed infants for further added oxidative damage at critical time period.

The American Academy of Pediatrics Committee on Nutrition recommends early use of iron-fortified formula instead of cow milk [27]. This has reduced the prevalence of anemia in children ages from 6 months to 60 months[35]. Since 1974, the Special Supplemental Nutrition Program for Women, Infants and Children (WIC) provides only high iron formula, unless there are special instructions against this by a health care provider. In 2001 the National Academy of Sciences recommended that the Dietary Reference Intake of iron (DRI) in infants 0-6 months to be 0.27 mg/day with a tolerable upper intake level of 40 mg/day with an assumption that 75% of iron is from heme iron sources [36]. Mature human breast milk contains 0.5 mg/L of iron [35], a considerably lower concentration of iron than iron-fortified baby formulas which also vary in the type and source of protein (e.g., bovine, soy or protein hydrolysate). The aim of this study is to explore whether the amount of iron consumed in the first 4 months of life is associated with T1DM before 6 years of age. A 6 year age cut off was used mainly because children are exposed to more environmental stimuli after that age. Even though a cause-effect relationship cannot be validated with this study, we aimed to assess a potential association between iron consumption in the first 4 months of life and risk of developing T1DM as a preliminary step for future studies.

## Materials and methods

### Subjects

This was a case-control study among children diagnosed with T1DM before the age of six and who at the time of recruitment were not older than 10 years and were receiving care from the pediatric diabetes clinic at the Birmingham Children's Hospital, a University of Alabama at Birmingham (UAB) affiliated hospital. Patients were identified through the Children's Hospital electronic medical records system using ICD 9 diagnosis codes 250.01, 250.03 and 250.13. Surveys were sent to a total of 250 children diagnosed with T1DM before 6 years of age and at the time of survey not >10 years old. A total of 141 surveys from children with T1DM were returned of which 130 children with diabetes were entered in the analysis and 11 excluded because of unclear answers. Some (n = 75) of the control questionnaires were rejected due to ambiguity in the answers leaving 83 controls eligible for further analysis. Thus our main sample consisted of a total of 130 T1DM cases and 83 controls which includes siblings of T1DM cases. Since type 1 diabetes is characterized by significant insulinopenia which requires insulin administration to control blood sugar, the controls were selected based on parents history of whether or not the sibling had diabetes. Siblings of T1DM children were included in the study if they were aged between 6 years and 10 years of age.

### Methods

After obtaining approval by the University of Alabama at Birmingham Institutional Review Board, 250 questionnaire surveys were mailed out between June 2006 and April 2007. *Data collection: *A questionnaire and letter of introduction stating that this research is being conducted to determine whether maternal nutrition, childhood infections, co-morbidities and the type and components of infant formula (e.g., minerals and vitamins) are associated with T1DM were mailed to the parents or guardians of the study participants. A questionnaire, which was completed by the parent or care-taker, was used to assess maternal medical history and baby feeding practices (e.g., gestational diabetes, maternal T1DM or type 2 diabetes (T2DM), period of exclusive breast feeding, introduction of supplementary infant formula, type of formula consumed in the first 4 months of life, number of weeks on formula, and vitamins and iron supplements) as well as child specific information such as birth weight, year of birth and illnesses in the first 4 months of life. The names of the patients and their caregivers were not included on the survey form. Anonymous filled questionnaires were returned to the Children's Hospital in self-addressed stamped return envelopes (without participants return addresses) provided by the researchers.

### Assessment of amount and duration of intake of specific iron containing foods

The infant formulas were then classified as high or low iron formula based on the self-reported formula brand name. The majority of available products at the time of the study were high iron except Similac low iron^®^, Similac PM 60/40^® ^and Enfamil Lipil low iron^® ^infant formulas. Comparisons were made between children with T1DM and non-diabetic siblings based on consumption of high-iron, low-iron formulas and breast milk.

If a survey was marked as formula fed for a month, we used a 30 day month. Calculation of total amount of formula ingested was based on normal caloric needs of food intake for infants [37]. Breast milk has 0.5 mg/L of iron, equivalent to 0.075 mg in 5 oz [35]. "High" iron formula has 12 mg/L (1.8 mg/5 oz) of iron and "low" iron formula has 4.6 mg/L (0.7 mg/5oz) of formula of ferrous iron. Table 1 depicts the expected amount of iron ingested based on the recommended daily intake of formula or breast milk in the first 4 months of life.

**Table 1:** The amount of iron intake that a normal term infant receiving the recommend daily allowance (RDA) of formula or breast milk would receive in the United States.*

Month	RDA milk (oz/day)	Iron, mg/day			Vitamin with iron (10 mg/serving)
			
		High-iron formula	Low-iron formula	Breast milk	
First month	23	8.28	3.22	0.345	300
Second month	29	10.44	4.06	0.435	300
Third month	29	10.44	4.06	0.435	300
Fourth month	30	10.8	4.2	0.45	300

* Breast milk has 0.5 mg/L of iron, equivalent to 0.074 mg in 5 oz [35]. High iron formula has 1.8 mg/5 oz of formula and low iron formula has 0.7 mg/5 oz of formula.

### Inclusion and exclusion criteria

We excluded individuals where the parent could not recall the type of infant nutrition source and whether or not solid foods (e.g., cereals) were introduced before 4 months of age, if there were serious illnesses prior to the diagnosis of diabetes (to exclude medication-induced diabetes), if gestational length was ≤ 37 weeks) and if diabetes was diagnosed after 6 years of age. Siblings were excluded from the study if they were >10 years at the study point for age- matching with the cases.

### Statistical analyses

Data management and analysis was done in Microsoft EXCEL^® ^and Statistical Analysis System (SAS version 9.2, SAS Institute Inc., Cary, NC). From total sample of 213, we excluded individuals who had missing data on age (n = 16), sibling rank (n = 1) and one child who iron intake was implausible (11,988 mg). Thus we had 195 children (67 controls and 128 cases) for the main analyses. For subgroup analyses, we created a subset of 59 controls and 59 cases where case had a control sibling. The distribution of iron intake and other continuous variables was checked for normality and log- or square root-transformed where appropriate to improve normality before comparing cases and controls. Because transformations did not completely correct the skewed nature of the data, we used the Wilcoxon Rank Sum test to test for the significance of differences in iron intake as well in other variables between cases and controls. Multivariable conditional logistic regression was performed to test whether the intake of iron is associated with T1DM before and after adjusting for birth weight, age at the time of the survey and birth order. In the final sample, there were two few mother's with a history of gestational diabetes or T2DM (< 4%). Thus these variables were not included in the multivariate conditional logistic analyses. The results are reported as odds ratios (OR) and 95% confidence intervals (CI) for 1 SD increase in iron intake for all sources. Associations were considered significant at a *P*-value of 0.05.

## Results

The mean (± SD) age at the time of diagnosis of T1DM was 3.23 ± 1.32 years. All study participants were born between 1996 and 2006. The general characteristics of the study population by case-control status are shown in Table 2. There was no association between sibling rank and T1DM, nor was there an association between maternal history of gestational diabetes or T2 D and T1DM in children.

**Table 2:** characteristics of the study participants by diabetes status.*

	Controls (n = 67)		Type 1 diabetes cases (n = 128)		
			
Variable	Median	Min, Max	Median	Min, Max	*P*
Age at diagnosis, y	--	--	3.0	0.16, 6.0	--
Age at recruitment, y	5.0	0.4,10.0	6.0	2.0, 10.0	0.05
Birth weight, kg	3.32	2.3, 4.7	3.32	1.11, 4.91	0.94
Total iron: form., suppl. & breast milk, mg	466	50, 1224	1159	50, 2399	< 0.0001
Iron from formula & supplements, mg	466	0, 1213	1144	0, 2399	< 0.0001
Duration of feeding low-iron formula, wks	12	0, 16	0	0, 16	< 0.0001
Duration of feeding high-iron formula, wks	12	0, 32	10	0, 16	0.10
Duration of breast feeding, wks	12	0, 16	4	0, 16	0.001
Mother had gestational diabetes, %	0		3		0.30
Mother has diabetes, %	3		4		1.00
Sibling rank, %					
First	42		49		0.44
Second	49		42		
Third	6		8		
Forth or higher	3		1		

* Values are medians, minimum, maximum or percentages.

†*P *values are from the Wilcoxon rank sum test for continuous variables and from the Fisher's exact test for categorical variables.

The median duration of breast feeding was significantly higher (*P *< 0.05) in controls (12 weeks) than in T1DM cases (4 weeks). The median (min, max) total iron intake in the first 4 months of life was 1159 (50, 2399) mg in T1DM cases and 466 (50, 1224) mg among controls (*P *< 0.001). The SD for iron intake was 540 mg in the total sample and 539 mg in the restricted sample. Table 3 depicts odds ratios for the association between 1 SD increase in total iron intake from all sources in first four months of life and type 1 diabetes. For each one standard deviation increase in iron intake, the odds ratio (95% confidence interval) for type 1 diabetes was 2.01 (1.183, 3.41) among all participants (128 cases and 67 controls) while it was 2.26 (1.27, 4.03) in a restricted sample of T1 D cases with a control sibling (59 cases and 59 controls) in models adjusted for birth weight, age at the time of the survey and birth order. In multivariate analyses, only age at the recruitment was associated with T1DM. For each one year increase in age, the odds ratio for T1DM was 1.44 (1.07, 1.95) in models adjusted for total iron intake, birth weight and sibling rank.

**Table 3:** Odds ratios (95% CI) for the association between total iron intake in first four months of life and type 1 diabetes.

	Odds ratios (95% CI) for 1 SD increase in iron intake*	
	**All participants** **(128 cases and 67 controls)**	**Restricted to cases with a control sibling** **(59 cases and 59 controls)**

Total iron intake, SD units†	2.01 (1.183, 3.41)	2.26 (1.27, 4.03)
Age at recruitment, y	1.44 (1.07, 1.95)	1.69 (1.15, 2.49)
Birth weight, kg	1.09 (0.37, 3.27)	0.77 (0.24, 2.51)
Sibling rank	1.46 (0.62, 3.41)	2.17 (0.77, 6.08)

* Odds ratios are from conditional logistic regression models in which the 4 continuous variables were modeled simultaneously.

† Total iron intake from formula, supplements and breast milk. In the total sample (n = 195) one SD unit was 540 mg while it was 539 mg in the restricted sample (n = 118).

## Discussion

The novel proposition of a causal link between the quantity of iron intake in the first 4 months of life and future development of T1DM is plausible considering the known strong association between ferritin and Type 2 diabetes in adults [38,39], a disorder sometimes diagnostically indistinct from T1DM with similar surrogate autoimmune markers and medical histories. Because in genetically susceptible subjects β-cell auto antibodies may materialize in infancy [40], this observation buttresses the argument that the environmental trigger for diabetes autoimmunity occurs early in life.

The physiological anemia of infancy seen in the first 2-3 months of life is not due to iron deficiency and does not respond to iron supplementation [35]. A normal term infant has sufficient iron stores for the first 4-6 months of life. Hence, the introduction of iron fortified formula in the critical period of 4-6 months could be potentially baneful. There has been a significant reduction in the prevalence of iron deficiency anemia in 6 to 60 month-old children after the introduction of iron-fortified baby formulas. However, there is limited clinical evidence which can be adduced to support the early introduction of iron fortified formulas in the first 4 months of life.

Although the mechanism remains unresolved, numerous studies have confirmed that prolonged breast feeding protects against T1DM [23], and breast feeding activists advocate the postponement of formula feeding. As regards to iron intake with breast feeding, even though 50% of the iron is absorbed, which is incontestably a high proportion when compared with cow's milk or iron fortified formula, breast milk per unit volume contains roughly 1/10^th ^of the iron content of the "low" iron formulas [27,32,33,35]. The net result is that breast fed babies absorb quantitatively far less iron than formula-fed babies, and this fact alone may explain the putative effect of breast milk.

Another confounding variable is that breast fed babies may delay feeding formula, cow's milk, cereals or other solid foods. This practice may avert exposure of the infant to foreign food antigens, such as gluten and bovine insulin, which may induce a cross-reacting immune response, particularly if gut permeability is altered by microbial infection [41]. Contrary to other studies [35], our study did not show that the number of weeks of breast milk ingestion conferred protection, most likely due to the small sample size in our study. Moreover, only a minority of children were on low-iron formula and the majority of children who had low total iron intake in the first 4 months had at some point also received breast milk. The absence of statistically significant association between the number of weeks of breast milk intake and having diabetes in our study is not enough to abandon the tenet that breast feeding is a protective factor against T1DM.

Type 1 diabetes involves the inexorable destruction of β cells secondary to autoimmunity. In iron overload conditions such as hemochromatosis, there is excessive iron storage in solid organs and selective destruction of β cells. Iron overload form hereditary hemochromatosis is associated with diabetes and impaired glucose tolerance and decreased insulin secretory capacity, which is reportedly reversed with phlebotomy therapy [42,43]. Excess pancreatic β-cells iron reportedly impairs mitochondrial function and glucose-stimulated insulin secretion [43,44].

The etiology of T1DM is likely multifactorial with a complex interplay between genetic, autoimmune, environmental, infectious and dietary factors. The role of iron in the infant formula in manifestation of T1DM was explored in this study. We could not find any studies examining relationship of iron status before onset of T1DM in genetically susceptible individuals. However, as in other autoimmune disorders, there are reports suggesting iron status may play a role in manifestation of systemic lupus erythematosis in murine models [45]. Iron is involved in the proliferation of T cells and other cells of immune system [46]. Similarly, ferritin reportedly has immune regulatory roles as well [47]. The pathoetiology by which fortified iron formulas could predispose to development of autoimmune diabetes in genetically susceptible individuals is uncertain. Whether the development of T1DM appertains to the actual quantity of iron ingested or is somehow attributable to the type of the fortified iron (ferrous sulfate vs. ferrous fumarate), or duration and timing of iron intake is likewise unclear. Excess cellular uptake of ferrous iron could in theory initiate a oxidation cascade which overwhelms the numerous intracellular protective anti-oxidation biochemical strategies [48]. It is thereby conceivable that the oxidative stress from immoderate iron exposure in the first 4 months in genetically susceptible infants may promote autoimmunity due to overproduction of free radical oxidative species. Parenthetically, ferrous fumarate is thought to produce minimal or no oxidative stress in vitro compared with ferrous sulfate [34]. And, interestingly, lactoferrin in breast milk reportedly attenuates iron-induced oxidative damage in vitro[34]. Finally, in the gut an overabundance of iron may modify the gastrointestinal immune response or its micronutrient milieu [49]. In conclusion, large-scale prospective studies are warranted to determine whether low iron or lactoferrin enriched formulas forefend against T1DM in high risk individuals.

### Limitations of the study

The major methodological pitfall of this retrospective study is that the data are self-reported. Hence, there is a potential chance of error insofar as this information relies on maternal dietary recall over a long duration. A memory bias may exist between that of the child with diabetes and non- diabetic sibling and that could have caused differential misclassification. Even though the letter accompanied the survey did not specify our hypothesis on iron content of formula, it mentioned we are investigating a link between T1DM and any of the minerals, vitamins, infant formulas or maternal nutrition. As the surveys were anonymous we do not have any data (clinical, demographic or medical) about the non-responders. We have excluded all ambiguous surveys. The choice of infant formula after birth depends on parental preferences, cost, co-morbid conditions like colic, acid reflux disease, lactose intolerance, milk protein allergy, type of formula the mother is first exposed too, etc. Consequently, the choice of low iron formula could be a proxy for socioeconomic characteristics or other factors which were not assessed in the surveys. Another confounding factor is that early introduction of bovine milk formula, besides increasing iron intake, may introduce previously proposed diabetogenic protein antigens.

## Conclusion

This pilot study suggests that higher intake of iron in the first 4 months of life is a probable risk factor for future development of T1DM. These results are not conclusive as this study was not designed to assess risk for T1DM. Even though the association of "high iron intake" with T1DM is seen in this study, in view of potential biases that may have confounded the results of this study, a future prospective large scale study to assess the possible association between high dietary intake of iron in infancy and T1DM is warranted. Whether iron intake is causal or a marker of another risk factor needs to be further investigated.

## Competing interests

The authors declare that they have no competing interests.

## Authors' contributions

AA contributed to the study conception, design, data interpretation, drafting and revising the manuscript. NBE conceived the study, participated in the design, data collection and analysis. EKK contributed to statistical analysis, interpretation, and revising the manuscript critically for important intellectual content. JH contributed to study design, study coordination, data collection and organization. SM involved in statistical analysis, interpretation and drafting of the manuscript. KM involved in the study conception, design, and critical revising of the manuscript for important intellectual content. All authors read and approved the final manuscript.

## References

[B1] Harjutsalo V Sjoberg L Tuomilehto J Time trends in the incidence of type 1 diabetes in Finnish children: a cohort study Lancet 2008 371 1777 1782 10.1016/S0140-6736(08)60765-5 18502302

[B2] Variation and trends in incidence of childhood diabetes in Europe. EURODIAB ACE Study Group Lancet 2000 355 873 876 10.1016/S0140-6736(99)07125-1 10752702

[B3] Karvonen M Viik-Kajander M Moltchanova E Libman I LaPorte R Tuomilehto J Incidence of childhood type 1 diabetes worldwide. Diabetes Mondiale (DiaMond) Project Group Diabetes Care 2000 23 1516 1526 10.2337/diacare.23.10.1516 11023146

[B4] Vehik K Hamman RF Lezotte D Norris JM Klingensmith G Bloch C Rewers M Dabelea D Increasing incidence of type 1 diabetes in 0- to 17-year-old Colorado youth Diabetes Care 2007 30 503 509 10.2337/dc06-1837 17327312

[B5] Hypponen E Kenward MG Virtanen SM Piitulainen A Virta-Autio P Tuomilehto J Knip M Akerblom HK Infant feeding, early weight gain, and risk of type 1 diabetes. Childhood Diabetes in Finland (DiMe) Study Group Diabetes Care 1999 22 1961 1965 10.2337/diacare.22.12.1961 10587826

[B6] Peng H Hagopian W Environmental factors in the development of Type 1 diabetes Rev Endocr Metab Disord 2006 7 149 162 10.1007/s11154-006-9024-y 17203405

[B7] Verge CF Howard NJ Irwig L Simpson JM Mackerras D Silink M Environmental factors in childhood IDDM. A population-based, case-control study Diabetes Care 1994 17 1381 1389 10.2337/diacare.17.12.1381 7882806

[B8] Siemiatycki J Colle E Campbell S Dewar RA Belmonte MM Case-control study of IDDM Diabetes Care 1989 12 209 216 10.2337/diacare.12.3.209 2702913

[B9] Thorsdottir I Birgisdottir BE Johannsdottir IM Harris DP Hill J Steingrimsdottir L Thorsson AV Different beta-casein fractions in Icelandic versus Scandinavian cow's milk may influence diabetogenicity of cow's milk in infancy and explain low incidence of insulin-dependent diabetes mellitus in Iceland Pediatrics 2000 106 719 724 10.1542/peds.106.4.719 11015514

[B10] Blom L Nystrom L Dahlquist G The Swedish childhood diabetes study. Vaccinations and infections as risk determinants for diabetes in childhood Diabetologia 1991 34 176 181 10.1007/BF00418272 1884889

[B11] Ziegler AG Schmid S Huber D Hummel M Bonifacio E Early infant feeding and risk of developing type 1 diabetes-associated autoantibodies Jama 2003 290 1721 1728 10.1001/jama.290.13.1721 14519706

[B12] Strotmeyer ES Yang Z LaPorte RE Chang YF Steenkiste AR Pietropaolo M Nucci AM Shen S Wang L Wang B Dorman JS Infant diet and type 1 diabetes in China Diabetes Res Clin Pract 2004 65 283 292 10.1016/j.diabres.2004.02.007 15331209

[B13] Wahlberg J Vaarala O Ludvigsson J Dietary risk factors for the emergence of type 1 diabetes-related autoantibodies in 21/2 year-old Swedish children Br J Nutr 2006 95 603 608 10.1079/BJN20051676 16578935

[B14] Paronen J Knip M Savilahti E Virtanen SM Ilonen J Akerblom HK Vaarala O Effect of cow's milk exposure and maternal type 1 diabetes on cellular and humoral immunization to dietary insulin in infants at genetic risk for type 1 diabetes. Finnish Trial to Reduce IDDM in the Genetically at Risk Study Group Diabetes 2000 49 1657 1665 10.2337/diabetes.49.10.1657 11016449

[B15] Borch-Johnsen K Joner G Mandrup-Poulsen T Christy M Zachau-Christiansen B Kastrup K Nerup J Relation between breast-feeding and incidence rates of insulin-dependent diabetes mellitus. A hypothesis Lancet 1984 2 1083 1086 10.1016/S0140-6736(84)91517-4 6150150

[B16] Gimeno SG de Souza JM IDDM and milk consumption. A case-control study in Sao Paulo, Brazil Diabetes Care 1997 20 1256 1260 10.2337/diacare.20.8.1256 9250450

[B17] Perez-Bravo F Carrasco E Gutierrez-Lopez MD Martinez MT Lopez G de los Rios MG Genetic predisposition and environmental factors leading to the development of insulin-dependent diabetes mellitus in Chilean children J Mol Med 1996 74 105 109 10.1007/BF00196786 8820406

[B18] Gerstein HC Cow's milk exposure and type I diabetes mellitus. A critical overview of the clinical literature Diabetes Care 1994 17 13 19 10.2337/diacare.17.1.13 8112184

[B19] Virtanen SM Knip M Nutritional risk predictors of beta cell autoimmunity and type 1 diabetes at a young age Am J Clin Nutr 2003 78 1053 1067 14668264 10.1093/ajcn/78.6.1053

[B20] Kaprio J Tuomilehto J Koskenvuo M Romanov K Reunanen A Eriksson J Stengard J Kesaniemi YA Concordance for type 1 (insulin-dependent) and type 2 (non-insulin-dependent) diabetes mellitus in a population-based cohort of twins in Finland Diabetologia 1992 35 1060 1067 10.1007/BF02221682 1473616

[B21] Hyttinen V Kaprio J Kinnunen L Koskenvuo M Tuomilehto J Genetic liability of type 1 diabetes and the onset age among 22,650 young Finnish twin pairs: a nationwide follow-up study Diabetes 2003 52 1052 1055 10.2337/diabetes.52.4.1052 12663480

[B22] Mayer EJ Hamman RF Gay EC Lezotte DC Savitz DA Klingensmith GJ Reduced risk of IDDM among breast-fed children. The Colorado IDDM Registry Diabetes 1988 37 1625 1632 10.2337/diabetes.37.12.1625 3192037

[B23] Sadauskaite-Kuehne V Ludvigsson J Padaiga Z Jasinskiene E Samuelsson U Longer breastfeeding is an independent protective factor against development of type 1 diabetes mellitus in childhood Diabetes Metab Res Rev 2004 20 150 157 10.1002/dmrr.425 15037991

[B24] Norris JM Scott FW A meta-analysis of infant diet and insulin-dependent diabetes mellitus: do biases play a role? Epidemiology 1996 7 87 92 10.1097/00001648-199601000-00015 8664407

[B25] Hummel M Fuchtenbusch M Schenker M Ziegler AG No major association of breast-feeding, vaccinations, and childhood viral diseases with early islet autoimmunity in the German BABYDIAB Study Diabetes Care 2000 23 969 974 10.2337/diacare.23.7.969 10895848

[B26] Couper JJ Steele C Beresford S Powell T McCaul K Pollard A Gellert S Tait B Harrison LC Colman PG Lack of association between duration of breast-feeding or introduction of cow's milk and development of islet autoimmunity Diabetes 1999 48 2145 2149 10.2337/diabetes.48.11.2145 10535447

[B27] Iron fortification of infant formulas. American Academy of Pediatrics. Committee on Nutrition Pediatrics 1999 104 119 123 10.1542/peds.104.1.119 10390274

[B28] Andrews NC Disorders of iron metabolism N Engl J Med 1999 341 1986 1995 10.1056/NEJM199912233412607 10607817

[B29] Iron balance and requirements in infancy Pediatrics 1969 43 134 142 5764062

[B30] McDonald MC Abrams SA Schanler RJ Iron absorption and red blood cell incorporation in premature infants fed an iron-fortified infant formula Pediatr Res 1998 44 507 511 10.1203/00006450-199810000-00007 9773838

[B31] Stekel A Olivares M Pizarro F Chadud P Lopez I Amar M Absorption of fortification iron from milk formulas in infants Am J Clin Nutr 1986 43 917 922 3717066 10.1093/ajcn/43.6.917

[B32] Saarinen UM Siimes MA Iron absorption from infant milk formula and the optimal level of iron supplementation Acta Paediatr Scand 1977 66 719 722 10.1111/j.1651-2227.1977.tb07978.x 579048

[B33] Hertrampf E Olivares M Pizarro F Walter T High absorption of fortification iron from current infant formulas J Pediatr Gastroenterol Nutr 1998 27 425 430 10.1097/00005176-199810000-00012 9779972

[B34] Raghuveer TS McGuire EM Martin SM Wagner BA Rebouche CJ Buettner GR Widness JA Lactoferrin in the preterm infants' diet attenuates iron-induced oxidation products Pediatr Res 2002 52 964 972 12438677 10.1203/00006450-200212000-00024

[B35] Wu AC Lesperance L Bernstein H Screening for iron deficiency Pediatr Rev 2002 23 171 178 10.1542/pir.23-5-171 11986493

[B36] Trumbo P Yates AA Schlicker S Poos M Dietary reference intakes: vitamin A, vitamin K, arsenic, boron, chromium, copper, iodine, iron, manganese, molybdenum, nickel, silicon, vanadium, and zinc J Am Diet Assoc 2001 101 294 301 10.1016/S0002-8223(01)00078-5 11269606

[B37] Jeannette Brakhane Endres RR Cynthia G Mense, Food, Nutrition, and the Young Child 2003 5 Prentice Hall

[B38] Jiang R Manson JE Meigs JB Ma J Rifai N Hu FB Body iron stores in relation to risk of type 2 diabetes in apparently healthy women Jama 2004 291 711 717 10.1001/jama.291.6.711 14871914

[B39] Forouhi NG Harding AH Allison M Sandhu MS Welch A Luben R Bingham S Khaw KT Wareham NJ Elevated serum ferritin levels predict new-onset type 2 diabetes: results from the EPIC-Norfolk prospective study Diabetologia 2007 50 949 956 10.1007/s00125-007-0604-5 17333112

[B40] Kimpimaki T Kupila A Hamalainen AM Kukko M Kulmala P Savola K Simell T Keskinen P Ilonen J Simell O Knip M The first signs of beta-cell autoimmunity appear in infancy in genetically susceptible children from the general population: the Finnish Type 1 Diabetes Prediction and Prevention Study J Clin Endocrinol Metab 2001 86 4782 4788 10.1210/jc.86.10.4782 11600541

[B41] Faria AM Weiner HL Oral tolerance: therapeutic implications for autoimmune diseases Clin Dev Immunol 2006 13 143 157 10.1080/17402520600876804 17162357 PMC2270752

[B42] Abraham D Rogers J Gault P Kushner JP McClain DA Increased insulin secretory capacity but decreased insulin sensitivity after correction of iron overload by phlebotomy in hereditary haemochromatosis Diabetologia 2006 49 2546 2551 10.1007/s00125-006-0445-7 17019598

[B43] McClain DA Abraham D Rogers J Brady R Gault P Ajioka R Kushner JP High prevalence of abnormal glucose homeostasis secondary to decreased insulin secretion in individuals with hereditary haemochromatosis Diabetologia 2006 49 1661 1669 10.1007/s00125-006-0200-0 16538487

[B44] Cooksey RC Jouihan HA Ajioka RS Hazel MW Jones DL Kushner JP McClain DA Oxidative stress, beta-cell apoptosis, and decreased insulin secretory capacity in mouse models of hemochromatosis Endocrinology 2004 145 5305 5312 10.1210/en.2004-0392 15308612

[B45] Leiter LM Reuhl KR Racis SP Jr Sherman AR Iron status alters murine systemic lupus erythematosus J Nutr 1995 125 474 484 7876923 10.1093/jn/125.3.474

[B46] Bowlus CL The role of iron in T cell development and autoimmunity Autoimmun Rev 2003 2 73 78 10.1016/S1568-9972(02)00143-X 12848962

[B47] Recalcati S Invernizzi P Arosio P Cairo G New functions for an iron storage protein: the role of ferritin in immunity and autoimmunity J Autoimmun 2008 30 84 89 10.1016/j.jaut.2007.11.003 18191543

[B48] Qian SY Buettner GR Iron and dioxygen chemistry is an important route to initiation of biological free radical oxidations: an electron paramagnetic resonance spin trapping study Free Radic Biol Med 1999 26 1447 1456 10.1016/S0891-5849(99)00002-7 10401608

[B49] Markel TA Crisostomo PR Wang M Herring CM Meldrum KK Lillemoe KD Meldrum DR The struggle for iron: gastrointestinal microbes modulate the host immune response during infection J Leukoc Biol 2007 81 393 400 10.1189/jlb.0906579 17255516

